# Supplementary material for: A Likelihood Approach for Real-Time Calibration of Stochastic Compartmental Epidemic Models
Source: PLoS Comput Biol. 2017 Jan 17;13(1):e1005257. doi: 10.1371/journal.pcbi.1005257 (PMC5240920; doi:10.1371/journal.pcbi.1005257)
Supplement: S1 File — (TAR.GZ) [file pcbi.1005257.s014.tar.gz › HSPH_Online-SI-Revision/EnKF07/n100-severe/n100-severe_PF2_EnKF-summary2.pdf]

peak -8	3w spec	3w cum	5w spec	5w cum	end	3w spec LOG	3w cum LOG	5w spec LOG	5w cum LOG	end LOG
mean	795.9	3486.1	97.	607.4	64.5	1.	1.1	1.5	0.7	0.6
median	491.7	2284.5	92.4	550.1	58.	0.9	1.2	1.4	0.7	0.4
quantile .1	87.1	78.2	67.5	82.5	44.6	0.6	0.2	0.7	0.5	0.2
quantile .9	2064.9	8243.6	136.	1257.9	89.8	1.4	1.8	2.6	0.5	1.2
27	14	23	15	37						

peak -4	3w spec	3w cum	5w spec	5w cum	end	3w spec LOG	3w cum LOG	5w spec LOG	5w cum LOG	end LOG
mean	91.7	93.6	94.8	82.6	80.9	1.8	0.7	2.6	0.9	1.
median	93.1	92.2	96.6	85.3	86.9	1.8	0.7	2.7	0.9	1.1
quantile .1	79.5	73.3	88.1	63.4	58.2	1.1	0.3	1.5	0.4	0.4
quantile .9	97.4	118.5	99.	98.1	91.	2.5	1.	3.3	0.4	1.3
4	25	0	17	14						

peak	3w spec	3w cum	5w spec	5w cum	end	3w spec LOG	3w cum LOG	5w spec LOG	5w cum LOG	end LOG
mean	95.2	90.7	94.6	90.1	89.4	1.3	0.9	1.6	0.9	0.9
median	94.6	91.9	94.6	91.3	91.	1.2	0.9	1.6	0.9	0.9
quantile .1	83.9	80.	75.3	78.8	77.5	0.9	0.6	0.9	0.6	0.6
quantile .9	106.1	98.4	114.9	97.5	97.	1.8	1.2	2.5	0.6	1.2
7	12	5	12	17						

peak +4	3w spec	3w cum	5w spec	5w cum	end	3w spec LOG	3w cum LOG	5w spec LOG	5w cum LOG	end LOG
mean	445.	417.7	1962.3	419.5	462.5	0.7	0.5	1.1	0.5	0.5
median	208.7	225.3	211.1	214.	237.9	0.6	0.5	0.9	0.5	0.5
quantile .1	88.6	120.8	84.	120.7	114.1	0.5	0.3	0.6	0.3	0.3
quantile .9	863.2	792.2	1567.7	886.8	1097.3	1.	0.7	1.8	0.3	0.8
47	34	42	35	35						

peak +8	3w spec	3w cum	5w spec	5w cum	end	3w spec LOG	3w cum LOG	5w spec LOG	5w cum LOG	end LOG
mean	205 406.	15 693.4	128 546.	18 308.1	20 916.2	1.6	1.2	1.8	1.2	1.3
median	2573.8	1943.9	2933.	2344.8	2730.3	1.3	1.1	1.5	1.1	1.2
quantile .1	142.2	269.7	97.9	233.2	228.	0.8	0.4	1.	0.4	0.4
quantile .9	130 030.	33 250.8	633 450.	41 888.2	46 347.5	2.9	2.2	3.2	0.4	2.3
19	5	21	6	6	{}	{}	{}	{}	{}	{}
